# Editorial to the Special Issue “Recent Advances in Optical Wireless Communications”

**DOI:** 10.3390/s26103098

**Published:** 2026-05-14

**Authors:** Borja Genoves Guzman, Máximo Morales Céspedes

**Affiliations:** Departamento Teoría de la Señal y Comunicaciones, Universidad Carlos III de Madrid, Av. de la Universidad 30, Leganés, 28911 Madrid, Spain; mmcesped@ing.uc3m.es

## 1. Introduction

The ever-increasing demand for wireless communication services has led to the search for alternative technologies. Recent progress in solid-state lighting, lasers, and detectors has propelled optical wireless communications (OWCs) to the forefront of future wireless technologies.

This Special Issue entitled “Recent Advances in Optical Wireless Communications” collects the latest breakthroughs in this evolving field, ranging from physical-layer enhancements to system-level applications. In alignment with the goals of this Special Issue, every contribution offers an exhaustive discussion and a robust bibliographical framework, meticulously detailing current trends and addressing the critical challenges inherent to their respective research.

## 2. Contributions

Paper [1] investigates a self-sustaining dual-hop decode-and-forward relaying architecture that utilizes simultaneous lightwave information and power transfer (SLIPT) to bridge the gap between energy efficiency and long-range link reliability. By employing a time-splitting technique, the relay harvests energy from the direct current component of the incident light to power its re-transmission, effectively creating an autonomous node suitable for remote 6G deployments. The authors develop a rigorous analytical framework using the Malaga distribution to characterize the optical links under realistic atmospheric turbulence and pointing errors, deriving closed-form expressions for outage probability, ergodic capacity, and bit error rate. Furthermore, the work provides a critical security evaluation by analyzing secrecy outage probability in the presence of an eavesdropper, demonstrating that SLIPT-based relaying not only facilitates sustainable communication but can also enhance the physical layer security of the network. This contribution is a significant milestone in the evolution of OWCs, as it offers a mathematically validated roadmap for implementing secure, energy-neutral backhaul solutions in future heterogeneous wireless ecosystems.

Paper [2] addresses the scalability of high-capacity optical networks by demonstrating a six-linear-polarized (LP)-mode strong-coupling mode-division multiplexing (MDM) transmission system utilizing intensity modulation and direct detection (IM/DD). To overcome the severe inter-modal crosstalk and modal delay characteristic of multi-mode fibers, the authors implement a sophisticated digital frequency-domain multiple-input multiple-output (MIMO) equalizer based on the least mean square algorithm. Their experimental approach showcases the successful transmission of a 36 GBaud OOK signal over 10 km of few-mode fiber, effectively managing the complexity of an MIMO architecture through frequency-domain processing. By proving the feasibility of strong-coupling MDM in cost-effective IM/DD systems, this work contributes a vital solution for the high-bandwidth, short-reach optical interconnects required by the next generation of data centers and 6G infrastructure.

Paper [3] presents an innovative framework for intelligent health monitoring by leveraging visible light communication (VLC) within 6G-enabled medical body sensor networks (MBSNs). To address the stringent reliability requirements of clinical environments, the authors utilize a site-specific Monte Carlo ray-tracing approach to characterize the optical propagation channel in a realistic hospital room, considering the impact of various medical equipment and mobility. Their experimental methodology integrates these channel models with machine learning (ML) techniques—specifically long short-term memory (LSTM) networks—to predict and enhance the quality of service (QoS) for vital sign monitoring. By demonstrating that VLC can provide a secure, interference-free, and high-capacity alternative to traditional radiofrequency (RF)-based sensor networks, this work contributes significantly to the evolution of OWC as a viable technology for safety-critical healthcare applications in the 6G era.

Paper [4] pushes the boundaries of high-capacity backhaul by demonstrating a 50 Gbps long-haul D-band radio-over-fiber (RoF) communication system spanning a distance of 4600 m. The research objective is to tackle the severe signal degradation caused by phase noise from laser linewidth and nonlinear distortions from optoelectronic devices, which typically limit the reach of photonics-aided terahertz systems. Their experimental methodology centers on the implementation of a novel two-dimensional convolutional neural network (2D-CNN) post-processing equalizer. By extracting intricate spatiotemporal features from preprocessed data, the 2D-CNN concurrently compensates for nonlinearities and phase noise, outperforming traditional Volterra series and deep neural network (DNN) methods. This work represents a major advancement in the evolution of OWC by proving that the integration of photonic-wireless technology with deep learning-based equalization can achieve the reliable, high-speed, and long-range connectivity essential for the ultra-broadband infrastructure of 6G networks.

The paper [5] addresses the critical challenge of efficient resource management in next-generation optical access networks by proposing a collaborative split learning (CSL)-based dynamic bandwidth allocation (DBA) scheme for 6G-grade time division multiplexing passive optical network systems. The objective is to enhance bandwidth utilization and reduce latency while preserving data privacy, which is often compromised in centralized artificial intelligence models. Their methodology involves partitioning a DNN between optical network units and the optical line terminal, allowing for collaborative model training without the need to share raw traffic data. Experimental results demonstrate that this CSL approach achieves superior prediction accuracy for bandwidth demand compared to traditional DBA methods, significantly lowering packet delay and jitter. By integrating privacy-preserving ML with optical resource allocation, this work contributes to the evolution of OWC and optical networking by providing a scalable, secure, and low-latency framework essential for the high-density traffic requirements of the 6G era.

Paper [6] enhances the robustness of optical camera communication (OCC) by developing a high-accuracy detection and classification model for color-shift keying (CSK) modulation. The primary objective is to overcome the performance limitations of traditional CSK detectors, which often struggle with signal distortion and noise in real-world environments. Their methodology introduces a deep learning (DL) approach, specifically utilizing a YOLOv8-based architecture to automate the detection of the light source and the classification of the modulated colors in a single step. Experimental results indicate that this DL-based model significantly outperforms conventional Euclidean distance-based methods, achieving near-perfect classification accuracy even at varying distances. By demonstrating that sophisticated computer vision techniques can effectively mitigate the physical-layer challenges of OCC, this work contributes to the evolution of OWC by providing a reliable and scalable framework for high-data-rate communication using standard camera sensors in Internet of Things and smart city applications.

Paper [7] demonstrates a high-capacity 82.5 GHz photonic W-band wireless transmission system, achieving record-breaking performance for OWC links. The research objective is to overcome the severe signal impairments—including nonlinearities—inherent in high-frequency IM/DD systems. Their experimental approach utilizes probability shaping 4-level pulse amplitude modulation (PS-PAM4) transmitted over 300 m, enabled by a hybrid equalization strategy that cascades a balanced, lightweight DNN with a clustering algorithm. This methodological combination allows for the effective mitigation of complex nonlinear distortions with reduced computational overhead compared to traditional models. By showcasing a reliable large data rate at the 82.5 GHz band, this work significantly contributes to the evolution of OWC by providing a practical and energy-efficient solution for the ultra-high-speed “last-mile” connectivity required in future 6G small-cell architectures.

Paper [8] proposes a novel cross-layer framework that integrates RF and OWC to meet the diverse QoS demands of 6G networks. The research objective is to optimize network efficiency through dynamic technology selection and modulation scheme selection, allowing the system to adapt in real time to varying channel conditions and application requirements. Their methodology utilizes a software-defined networking approach to manage the handover between RF and OWC links while selecting the most appropriate modulation—such as OFDM-IM for RF or DCO-OFDM for OWC—based on several performance metrics. By demonstrating significant improvements in spectral efficiency and system reliability through this hybrid architecture, the work contributes to the evolution of OWC by providing a scalable strategy for its seamless integration into the broader 6G heterogeneous landscape.
